# Sense of Purpose in Life and Escape from Self as the Predictors of Quality of Life in Clinical Samples

**DOI:** 10.1007/s10943-014-9833-3

**Published:** 2014-02-14

**Authors:** Magdalena Błażek, Maria Kaźmierczak, Tomasz Besta

**Affiliations:** Institute of Psychology, University of Gdansk, Bazynskiego 4a, 80-952 Gdańsk, Poland

**Keywords:** Depression, Suicide, Quality of life, Purpose in life

## Abstract

Depression is a leading mental disorder from which suffer Europeans and especially women. In clinical groups with elevated risk of suicidal tendencies, both the negative factors and psychological variables that can protect a person should be analyzed. The aims of the current study were analysis of purpose in life function in perceived quality of life—self-efficacy and life satisfaction among people suffering from depression in comparison with control group and analysis of escape from self (EfS)-function as an indicator of suicidal thoughts occurrence, for suicide attempt and perceived quality of life (life satisfaction). Two studies were conducted on two clinical groups. The first study consists of females, 20 of them with depression and 40 without depression disorder as a control group. Measures used in this study are Purpose in Life Scale, General Self-Efficacy Scale, and Satisfaction with Life Scale (SWLS). The second study consists of 60 participants, including 20 who attempted suicide. Measures used in this study are SWLS Scale and an EfS measure. There is a significant meaning of the sense of purpose of life for well-being and self-efficacy. The ability to maintain the feeling of sense of one’s existence seems to be a significant factor that protects from a decrease in life quality and keeps the feeling of being able to deal in difficult situations, as well as helps to accept depression symptoms.

## Introduction

Depression is a universal disorder, and among most cultures, there are high rates of both its presence at current point and throughout the whole life (Hammen 2006). World Health Organization (WHO) states that depression is a leading mental disorder from which suffer Europeans and especially women. In Poland, about million and a half people suffer from depression (Kwiatkowska 2007). Half of the people suffering from depression experience suicidal thoughts, and every tenth person commits suicide (Yapko 1999). The assessment is that in 2020, depression will be the number one cause of death (Murray and Lopez 1996).

According to WHO, in Europe today, the average suicide rate is 13.9 per 100,000 people. The highest rates are in Lithuania (30.7), Hungary (21.5), Finland (18.5), and Slovenia (18.4) (WHO 2011a). WHO data for Poland, where the studies were carried out, from 2008 indicate that suicide rate is 14.9 per 100,000 people and that men commit suicide more often (rate for men was 26.4, and for women, it was 4.1) (WHO 2011b).

Clinical approach (ICD—10, 2000) defines suicide as a deliberate act of self-mutilation. Especially, prone to auto-destruction are people with borderline disorder, hysteria, depression or with somatic disorders (Bhatia and Bhatia 2007; Pruitt 2007, Guirguis-Blake et al. 2008; Giffin 2008). Baumeister (1991) defines suicide as a form of escape from self (EfS); firstly, an individual looses positive feelings toward oneself and the sense of being worthy that leads to self-blame for failures and deteriorating life situation. People who try to commit suicide think that the crisis can be overcome by avoiding self-awareness that leads to loss of self-regulation (Baumeister et al. 1994).

Heisel and Flett (2004) point out that in clinical groups with elevated risk of suicidal tendencies, both the negative factors and psychological variables that can protect a person should be analyzed. The authors claim that suicidal tendencies can be a consequence of negative psychological factors (e.g., depression, neurotism, or sense of hopelessness) as well as a lack of positive ones. On the basis of their own research on psychiatric patients, Heisel and Flett stress the importance of sense of purpose in life which acts as a buffer against suicidal tendencies (see Wang et al. 2007). Although a literature review supported spirituality as coping method among individuals experiencing chronic stress (e.g., Kaye and Raghavan 2002; Ballew et al. 2011), having life goals is a special way to decrease the risk of suicidal thoughts (Lapierre et al. 2007). What is more, the protective function of sense of purpose in life exceeds the importance of life satisfaction and increases among patients with depression. Such conclusion confirms the credibility of negative associations between inclination toward depression and suicide tendencies that was found among nonclinical groups (e.g., Harlow et al. 1986; Kinnier et al. 1994; Wang et al. 2007). Sense of purpose in life is not only a factor that decreases the risk of suicidal tendencies but also addictions (e.g., Harlow et al. 1986; Kinnier et al. 1994; Marsh et al. 2003).

Particular value of the current study is the presence of clinical groups (that suffer from depression and that have attempted suicide) of different age. Two main aims of the current studies were identified:

1. Analysis of self-efficacy function in perceived quality of life—self-efficacy and life satisfaction among people suffering from depression in comparison with control group.
2. Analysis of EfS function as an indicator of suicidal thoughts occurrence, for suicide attempt and perceived quality of life (life satisfaction).

## Study 1

### Participants and Procedure

The participants were all female, 20 of them with depression (Age *M* = 38; *SD* = 10.6) and 40 without depression disorder as a control group (Age *M* = 35; *SD* = 10.5). Participants filled out questionnaires that included following scales: Purpose in Life Scale (PIL; Crumbaugh and Maholick 1964)—the PIL is a 20-item measure of existential meaning and purpose in life; General Self-Efficacy Scale GSES (Schwarzer et al. 1997)—this is a short, 10-item scale that measures the power of general perception of one’s efficacy when dealing with difficult situations and obstacles; Satisfaction with Life Scale, SWLS (Diener et al. 1985)—participants assess how each of the statements is relevant to their current life. Participants have 7 answers to chose from the following: from 1—I completely disagree to 7—I completely agree. The higher the score, the higher the life satisfaction. Acceptance of Illness Scale (AIS) (Felton, Revenson, and Hinrichsen, in Polish adaptation Juczyński 2009) consists of eight items related to consequences of bad health condition.

## Results

In line with predictions, significant differences emerged between control and experimental groups for life satisfaction (*M* = 23.9 and *M* = 11.3, respectively) *F* (1,58) = 131.817; *p* < .001; self-efficacy (*M* = 30.7 and *M* = 21.9, respectively) *F*-Brown-Forsythe (1,28) = 39.987; *p* < .001; and for sense of purpose in life (*M* = 110.2 and *M* = 63.1, respectively) *F* (1,58) = 120.677; *p* < .001.

Correlation analysis that was carried out between the measures, separately for the control and depression groups, showed a relationship between the sense of purpose in life and quality of life (both groups), efficacy (both groups), and illness acceptance (participants suffering from depression) (Table 1).

**Table 1:** Correlation analysis (for control group above the diagonal, for group with depression are below the diagonal)

	PIL	GSES	SWLS	AIS
PIL	–	.543**	.681**	na
GSES	.744**	–	.460**	na
SWLS	.666**	.740**	–	na
AIS	.666**	.683**	.573**	–

** *p* < .01

Two stepwise regression analyses were carried out: first one with the sense of life satisfaction as an outcome variable (Table 2); second one with self-efficacy as an outcome variable. The predictors put in the first step of regression analysis were age, education (dummy coded: higher education = 1), and marital status. In the second step, the type of group was introduced (dummy coded: depression = 1), and in the third step, PIL was introduced.

**Table 2:** Stepwise regression analysis; outcome variable: quality of life (*N* = 60)

	*R* ^*2*^ *adj.*	*F*	*Β*	*t*
Step 1	−.012	.767		
Age			−.160	−1.005
Education			.135	1.013
Single/in relationship			.125	.790
Step 2	**.674**	31.011***		
Age			−.009	−.098
Education			.068	.900
Single/in relationship			.081	.899
Group			−**.825**	−10.812***
Step 3	**.813**	51.469***		
Age			−.010	−.141
Education			.040	.711
Single/in relationship			.109	1.596
Group			−**.315**	−3.202**
PIL			**.631**	6.413***

Significant results are in bold

** *p* < .01; *** *p* < .001; *R* ^2^ change .657 for step 2, *p* < .001; and .133 for step 3, *p* < .001

The sense of purpose in life turned out to be a significant predictor of sense of life satisfaction (wellbeing), stronger one than being a part of the group suffering from depression. A significant change in *R* ^2^ after adding PIL score shows that this variable independently explains the additional variance of the wellbeing variable (Table 2).

Similar stepwise regression analysis with self-efficacy as an outcome variable confirmed predictive power of PIL. In third step of the regression, when all the variables were introduced, *Β* for PIL was .814 (*t* = 6.864; *p* < .001), while *Β* for group (depression vs. control) was insignificant *Β* = − .004; (*t* = − .036; *p* = in.). Once again, a significant change in *R* ^2^ after adding PIL score was observed (*R* ^*2*^ adj. for step 2 (without PIL) = .497; *R* ^2^ change = .220, *p* < .001 and *R* ^*2*^ adj. for step 3 (with PIL) = .729). When controlling for the sense of purpose in life, being a member of group with depression is no longer a significant predictor of self-efficacy level (see Table 3).

**Table 3:** Stepwise regression analysis; outcome variable: self-efficacy (*N* = 60)

	*R* ^*2*^ *adj.*	*F*	*Β*	*t*
Step 1	.060	2.238		
Age			−**.369**	−2.411*
Education			.092	.716
Single/in relationship			.219	1.438
Step 2	**.497**	15.328***		
Age			−**.248**	−2.189*
Education			.038	.407
Single/in relationship			.183	1.645
Group			**−.662**	−6.983***
Step 3	**.729**	32.154***		
Age			−**.249**	−2.992**
Education			.003	.045
Single/in relationship			**.22**	.677**
Group			−.004	−.036
PIL			**.814**	6.864***

Significant results are in bold

** *p* < .01; *** *p* < .001; *R* ^2^ change .423 for step 2, *p* < .001; and .220 for step 3, *p* < .001

## Study 2

### Participants and Procedure

Sixty participants took part in this study, among them a group of 20 people who attempted suicide (9 women). Mean age was 27.8 (*SD* = 7.1). A control group consisted of people (*N* = 40, 14 women) who did not attempt to commit a suicide. Participants filled out a questionnaire with demographics and questions concerning their suicide attempts as well as a SWLS Scale and an EfS measure that consists of three questions based on the EfS theory by Roy Baumeister (Błażek et al. 2008). Questions measure following variables: ability to evaluate the level of sense of purpose in life, control over one’s life, and self-awareness avoidance. Participants answered questions on a 7-point scale with 1—I completely disagree to 7—I completely agree.

## Results

As expected, large differences emerged between control group and the group with suicide attempts for satisfaction with life (*M* = 21.5 and *M* = 14.8, respectively) *F* (1,58) = 20.723; *p* < .001; and with the EfS levels (*M* = 17.3 and *M* = 13.1, respectively) *F*-Brown-Forsythe (1,25) = 13.547; *p* < .001. EfS was highly correlated with the sense of satisfaction with life among the participants from the suicide attempts group (*r* = .626; *p* < .01) but not in the control group (*r* = .170; *p* not significant).

Stepwise regression analysis was also carried out with satisfaction with life as an outcome variable. The predictors entered in the first step of regression analysis were age, sex, education (dummy coded: higher education = 1), and the number of family members. In the second step, the type of group was entered (dummy coded: suicide attempt = 1), and in the third step, the EfS scale was entered (see Table 4).

**Table 4:** Stepwise regression analysis; outcome variable: sense of life satisfaction (*N* = 60)

	*R* ^*2*^ *adj.*	*F*	*Β*	*t*
Step 1	**−.046**	**.350**		
Age			−.047	−.354
Sex			.089	.664
Education			.115	.862
No of family members			.038	.283
Step 2	**.235**	4.633***		
Age			−.037	−.321
Sex			.047	.411
Education			.019	.167
No. of family members			.15	1.279
Group			−**.548**	−4.61***
Step 3	**.335**	5.951***		
Age			−.037	−.345
Sex			.02	.19
Education			.015	.141
No of family members			.152	1.396
Group			**−.364**	−2.882**
Escape from self			**.372**	3.012**

Significant results are in bold

** *p* < .01; *** *p* < .001; *R* ^2^ change .275 for step 2, *p* < .001; and .102 for step 3, *p* < .01

Stepwise regression analysis with life satisfaction as an outcome variable confirmed predictive power of EfS scale. In third step of the regression, when all variables were introduced, *Β* for EfS was .372 (*t* = 3.012; *p* < .01). A significant change in *R* ^2^ after adding EfS score was observed (*R* ^*2*^ adj. for step 2 (without EfS) = .235; *R* ^2^ change = .102, *p* < .01 and *R* ^*2*^ adj. for step 3 (with EfS) = .335). Even when controlling for being a member of group with suicide attempt, EfS independently explains the additional variance of satisfaction with life variable.

## Discussion

The results obtained in the first study show a significant meaning of the sense of purpose in life for satisfaction measures (wellbeing) and for self-efficacy. The ability to maintain the feeling of sense of one’s existence seems to be a significant factor that protects from a decrease in life quality and keeps the feeling of being able to deal in difficult situations, as well as it helps to accept depression symptoms, which in turn allows to dealing with them in a more efficient manner.

The obtained results can thus be a base for therapeutic action for people who suffer from depression, which is, as stated by Harris and Barraclough (1997), the primary factor responsible for suicide attempts. A number of studies (see: Longvist 2000; Isacsson 2000) show that for a large number of suicide cases, depression was recognized. As conducted by the authors, studies on people who attempted suicide show the level of sense of meaning of life measured as the extent of EfS (research carried out according to the model suggested by Baumeister 1991) is the strongest predictor of life quality understood as well-being. Consequently, it can be said that the power to keep the feeling of purpose in life through ability to create goals, to make sense of everyday actions, and the feeling of control over one’s life are again significant factors that increase the overall quality of life and as a result protect from attempts at suicide.

The conducted research shows a significant role of the sense of purpose in life in constructing individual’s life. As Auhagen (2000) pointed out, meaning of life is strongly connected to well-being. The feeling of purpose of one’s existence is associated with better strategies of dealing with difficult, sometimes tragic experiences, which is a factor that protects from depression symptoms and suicide attempts.
